# Comparative analysis of bias and quality in YouTube videos on bariatric surgery and weight-loss medications

**DOI:** 10.1007/s00464-026-12795-5

**Published:** 2026-04-13

**Authors:** Julia Botvinov, Mason Henrich, Qichen Deng, Aram Jawed, Aziz M. Merchant

**Affiliations:** 1 Department of Surgery, Hackensack Meridian School of Medicine, Nutley, NJ USA; 2 Department of Surgery, Center for Weight Loss & Metabolic Health, John F. Kennedy University Medical Center, 65 James Street, 3rd Floor, Edison, NJ 08820 USA

**Keywords:** YouTube, Bariatric surgery, Weight-loss medications, GLP-1 agonists, Video quality, Patient education

## Abstract

**Background:**

Weight-loss surgery (WLS) remains the most effective treatment for clinically severe obesity, while weight-loss medications (WLM), particularly GLP-1 agonists, have recently gained widespread public attention. With YouTube serving as a major source of health information, the quality and tone of videos discussing these interventions warrant evaluation. This study compares the source, content quality, and perceived tone of YouTube videos related to WLS and WLM.

**Methods:**

Using an incognito browser, the first 200 “most relevant” YouTube videos for the search terms “weight loss surgery” and “weight loss medicine” were screened in October 2023. Eligible videos were categorized by source and content type. Video quality was assessed using the DISCERN instrument, and tone was classified as positive, neutral, or negative. Two independent reviewers scored all videos, with a third reviewer resolving disagreements. Descriptive statistics and nonparametric tests were used for comparisons, with statistical significance set at p < 0.05.

**Results:**

A total of 129 WLS and 111 WLM videos were analyzed. WLM videos demonstrated significantly higher view ratios (*p* < 0.0001) and more comments (*p* = 0.0014). WLM videos were predominantly commercial (76%) and informational (97%). Mean DISCERN scores were higher for WLM videos (46.69 ± 8.75; fair) compared with WLS videos (40.32 ± 8.70; poor; *p* = 0.0366). In contrast, WLS videos more frequently originated from academic centers (50%) and included patient experience content (49%). Tone differed significantly between groups (*p* < 0.01): 60% of WLS videos were positive, whereas WLM videos exhibited fewer positive (44%) and more negative tones (14%).

**Conclusions:**

WLM videos were of higher informational quality but conveyed a less positive overall tone, whereas WLS videos presented more positively but were of lower quality. These findings highlight substantial variation in publicly accessible YouTube content on weight-loss interventions and underscore the need for improved, high-quality patient education materials.

Weight loss surgery (WLS) is the most effective treatment for clinically severe obesity [1]. Long-term data support its durability, with patients who undergo sleeve gastrectomy or gastric bypass achieving total weight loss of 58.3% and 56.7%, respectively, after 10 or more years [2]. More recently, weight loss medicine (WLM) has emerged as an alternative treatment option. Glucagon-like peptide-1 (GLP-1) agonists, originally developed for type II diabetes mellitus, are now widely used for weight management [3]. GLP-1 analogs delay gastric emptying, prolonging satiety [3] and activation of central GLP-1 receptors contributes to decreased appetite [4].

The popularity of GLP-1 agonists has grown rapidly in recent years, influenced in part by celebrities and social media personalities [5]. This trend reflects a broader pattern of individuals turning to online platforms for health information, with 58% of internet users reporting that they browse health-related content online [6]. As one of the largest video-sharing platforms globally, YouTube has become a prominent source of health information for the general public. Because online content often mirrors cultural and clinical trends at a given moment, videos uploaded during periods of heightened interest in specific treatments may particularly influence public perception.

Previous studies have assessed the quality of YouTube videos related to WLS. Analyses of laparoscopic sleeve gastrectomy videos have shown that many are low-quality and unreliable [7, 8], while another study reported that 53.7% of bariatric surgery videos are useful, with most uploaded by physicians or hospitals [9]. To date, no study has compared the quality and perceived tone of YouTube videos on WLS and WLM [10].

The purpose of this study is to evaluate YouTube videos related to WLS and WLM and compare their source characteristics, informational quality, and perceived tone. Understanding how these treatment modalities are portrayed online may help identify potential biases in publicly accessible content and highlight opportunities to improve the quality of patient-facing educational material.

## Materials and methods

### Study design and video selection

This study followed STROBE guidelines for observational research. The YouTube video library was analyzed on October 22-23, 2023, using an incognito browser to prevent algorithmic personalization from influencing search results [11]. Two search queries, “weight loss surgery” and “weight loss medicine”, were entered, consistent with established methods for extracting YouTube content in prior studies [12–14]. To better reflect how a typical patient might access YouTube, searches were performed from a residential internet connection in New Jersey rather than from an academic institution, reducing potential bias related to institutional networks. Using an incognito browser and non-institutional network minimized personalization bias in search results.

Search results were filtered by relevance, YouTube’s default setting. The first 200 most relevant videos for each search term were recorded, and duplicates were removed. Exclusion criteria included non-English videos, audio-only videos, videos without audio, videos longer than one hour, and videos requiring sign-in to access. For “weight loss surgery,” videos were included if they provided an explanation of, commentary on, or opinion about sleeve gastrectomy, gastric bypass, duodenal switch, or gastric banding. For “weight loss medicine,” videos were included if they discussed glucagon-like peptide-1 (GLP-1) agonists for weight loss. Because YouTube content often reflects cultural and clinical trends at a given moment, videos uploaded during this period of heightened public interest in GLP-1 agonists were considered appropriate for capturing patient-facing information. A formal sample size calculation was not applicable; selecting the first 200 most relevant videos per search term follows standard YouTube study methodology.

### Video characteristics and categorization

For each video, the following characteristics were recorded: title, URL, duration (minutes), number of views, days since upload, view ratio (views per day), number of likes, and number of comments. Videos were categorized into five uploader types based on previously established methods [13]: Physician, Nonphysician, Patient, Commercial, and Academic. Physician videos were created by physicians or physician groups. Nonphysician videos were created by healthcare professionals such as physical therapists, physician associates, nurses, nutritionists, pharmacists, psychologists, and dietitians. Patient videos were created by individuals describing personal experiences. Commercial videos were produced by news outlets or companies, even when physicians appeared in the content, as the physicians were not responsible for uploading the material. Academic videos originated from individuals affiliated with academic medical centers, hospitals, or research groups.

Videos were also categorized by content type: Patient Experience, Advertisement, Instructional, or Informational. Patient Experience videos described personal accounts of undergoing the intervention. Advertisement videos promoted the intervention. Instructional videos explained the surgical technique or provided medication-use directions. Informational videos offered general education about the intervention.

### Quality and tone assessment

Video quality was assessed using the DISCERN instrument, a 16-item tool developed by the University of Oxford for evaluating consumer health information [15]. Each item is scored from 1 (worst) to 5 (best), yielding total scores from 16 to 80. Scores were categorized as “very poor” (16-28), “poor” (29-41), “fair” (42-54), “good” (55-67), or “excellent” (68-80). Two authors (JB and MH) independently scored each video. The two scores were then averaged to produce the final DISCERN score. All videos were evaluated using identical scoring procedures and definitions across both WLS and WLM groups to ensure consistency and comparability.

Tone was assessed using a system adapted from Keelan et al. and Bert et al. [16, 17]. Videos were classified as positive, negative, or neutral. Positive videos used supportive language about the intervention, encouraged its use, or refuted concerns about safety. Negative videos used discouraging language, questioned safety or efficacy, or presented the intervention as dangerous. Neutral videos provided information without clear positive or negative framing. Disagreements between the two reviewers (JB and MH) were resolved by a third reviewer (QD), who served as arbiter.

### Statistical analysis

Statistical analyses were performed using GraphPad Prism 10. Descriptive statistics were calculated for all video characteristics, DISCERN scores, and tone classifications. Continuous variables were summarized as mean ± standard deviation (SD). Between-group comparisons were made using the Mann-Whitney U test for nonparametric data, two-way ANOVA for multivariable comparisons, and the Kruskal-Wallis test for comparisons across more than two groups. A *p*-value < 0.05 was considered statistically significant. No missing data were present for any recorded variable. Analyses accounted for the cross-sectional nature of the sample, and no weighting or adjustment for sampling strategy was required.

### Ethics and registration

This study analyzed publicly available online content and did not involve human subjects; therefore, institutional review board approval and informed consent were not required. No study registration was applicable. Artificial intelligence-assisted editing tools were used for language refinement. The authors validated all text and are fully responsible for the work presented.

## Results

In total, 111 WLM and 129 WLS videos were included in the final analysis. The remaining video characteristics are outlined in Table 1. All mean values are reported as mean ± standard deviation. The mean duration for WLM and WLS videos was 5.10 ± 3.55 and 7.40 ± 14.57 min, respectively. The mean number of views for WLM and WLS videos was 142,573.61 ± 347,543.64 and 81,497.03 ± 222,559.81, respectively. The remaining video characteristics are outlined in Table 1. Shapiro-Wilk testing was performed for the video characteristics data, revealing a non-Gaussian distribution. Therefore, Mann-Whitney tests revealed significant differences between the two categories: WLM had a significantly higher view ratio (*p* < 0.0001) and number of comments (*p* = 0.0014). When categorized by source, out of 111 WLM videos, 84 (75.5%) were uploaded commercially, while out of 129 WLS videos, 65 (50.4%) were uploaded academically. The full breakdown of uploader data is in Table 2. The video content for WLS videos was roughly split, with 63 (48.8%) categorized as patient experience and 61 (47.3%) categorized as informational, while for WLM videos, 108 (97.3%) were informational. The remaining data for this metric can be found in Table 3.

**Table 1:** Video characteristics for WLS and WLM videos

Characteristic	WLS (Mean ± SD)	WLM (Mean ± SD)
Video duration (minutes)	7.4 ± 14.57	5.1 ± 3.55
Number of views	81 497.03 ± 222 559.81	142 573.61 ± 347 543.64
Days since upload	1610 ± 1162.74	454.3 ± 512.12
View ratio (views per day)	65.91 ± 227.85	455.86 ± 1,218*
Number of likes	784.07 ± 2047.46	2 260.93 ± 8 332.81
Number of comments	129.68 ± 456.23	297.47 ± 735.4*

^*^Statistically significant difference between WLS and WLM

*WLS* weight loss surgery, *WLM* weight loss medicine, *SD* standard deviation

**Table 2:** Video sources for WLS and WLM videos

Source	WLS (n, %)	WLM (n, %)
Physician	27 (20.9%)	21 (18.9%)
Non-physician	1 (0.8%)	2 (1.8%)
Patient	8 (6.2%)	0 (0%)
Commercial	28 (21.7%)	84 (75.7%)*
Academic	65 (50.4%)	4 (3.6%)
Total	129 (100%)	111 (100%)

^*^Statistically significant difference.

*WLS* weight loss surgery, *WLM* weight loss medicine

**Table 3:** Video content for WLS and WLM videos

Content type	WLS (*n*, %)	WLM (*n*, %)
Patient experience	63 (48.8%)	3 (2.7%)
Advertisement	0 (0%)	0 (0%)
Instructional	5 (3.9%)	0 (0%)
Informational	61 (47.3%)	108 (97.3%)
Total	129 (100%)	111 (100%)

*WLS* weight loss surgery, *WLM* weight loss medicine

The average DISCERN score for the WLM videos was 46.69 (SD = 8.75), which is considered “fair”. When stratified by video source, mean DISCERN scores were in the “good” category for physician-created videos, while “fair” in all other remaining categories. Additionally, there is a statistically significant difference between the DISCERN score for physician and commercial videos (*p* < 0.0001). The average score for WLS was 40.32, which is considered “poor". When stratified by video source, mean DISCERN scores were also all in the “poor category” and there was no significant difference in scoring between the video source types. The remaining data regarding DISCERN scoring is seen in Table 4. The average score for WLM videos was significantly higher than WLS videos (*p* = 0.0366).

**Table 4:** Average DISCERN score per video source

Source	WLS (Mean ± SD)	WLM (Mean ± SD)
Physician	40.57 ± 5.93	55.17 ± 6.83**
Non-physician	37.5 ± 0	48.25 ± 48.25
Patient	37.38 ± 8.52	n/a
Commercial	40.63 ± 6.85	44.55 ± 7.98**
Academic	40.51 ± 10.4	46.25 ± 7.97
All Videos	40.32 ± 8.7*	46.69 ± 8.75*

^*^Statistically significant overall difference.

^**^Significant difference between physician and commercial videos within WLM. *WLS* weight loss surgery, *WLM* weight loss medicine, *SD* standard deviation

The tone scoring for both WLM and surgery videos is shown in Table 5. For WLS videos, there were 78 (60%) positive videos, 46 (36%) neutral videos, and five (4%) negative videos. For WLM videos, there were 49 (44%) positive videos, 46 (41%) neutral videos, and 16 (14%) negative videos. Tone scoring differed significantly between the two categories (x^2^ = 11.10, df = 2; *p* < 0.01).

**Table 5:** Percentage of each tone score

Tone score	WLS (*n*, %)	WLM (*n*, %)
Positive	78 (60.5%)	49 (44.1%)
Neutral	46 (35.7%)	46 (41.4%)
Negative	5 (3.9%)	16 (14.4%)
Total	129 (100%)	111 (100%)

*WLS* weight loss surgery, *WLM* weight loss medicine

## Discussion

This study addressed a gap in the literature by comparing the quality and perceived tone of YouTube videos on WLS and WLM. We found substantial differences between the two categories in terms of uploader source, content type, tone, and overall informational quality. Most WLS videos originated from academic institutions and contained a mix of informational and patient experience content, whereas WLM videos were predominantly informational and uploaded by commercial entities. WLS videos demonstrated a more positive tone but lower DISCERN scores, while WLM videos had a higher proportion of neutral and negative tone videos, yet demonstrated higher overall quality. These findings highlight the diversity of messaging patients encounter online when seeking information about weight-loss treatment options.

WLM videos received significantly higher DISCERN scores than WLS videos, and several factors may explain this difference. First, the DISCERN instrument inherently favors videos that provide comprehensive, structured information on treatment mechanisms, benefits, risks, and alternatives. Prior studies have noted that informational videos tend to score higher than anecdotal or personal experience videos [8, 18–21]. Nearly all WLM videos were informational, whereas WLS videos were almost evenly split between informational and patient experience content, making lower scores among WLS videos expected. Second, because many WLM videos were produced by news outlets or commercial entities, the inclusion of legally required risk statements or disclaimers may have increased completeness and balance, thereby improving DISCERN scores [22].

Tone may also influence DISCERN scoring patterns. Although DISCERN is not designed to measure sentiment, videos with neutral tone may unintentionally align well with the instrument’s emphasis on balance, completeness, and discussion of both benefits and risks. In our study, neutral videos tended to have higher DISCERN scores. This observation aligns with prior findings showing no significant association between tone and DISCERN score [23], although additional research is needed to clarify this relationship, especially in the context of evolving online health communication.

Regarding tone differences between WLS and WLM videos, WLS content exhibited a much more positive tone. This may be attributable to the high proportion of patient experience videos in the WLS category; satisfied patients may be more likely to share their stories online, producing inherently positive messaging. In contrast, WLM videos were largely created by commercial sources rather than patients, reducing the presence of personal testimonials and increasing the likelihood of neutral or negative framing, such as discussion of risks, controversies, or side effects.

Uploader source may also shape tone and content. Over half of WLS videos were produced by academic institutions, which often use social media platforms for education and outreach [24–26]. With YouTube specifically, one study demonstrated that a physician’s educational video on myelofibrosis resulted in more than a 150% increase in new patient visits to his clinic within six years of the video’s upload compared with a clinic that did not have a corresponding video [25]. This highlights the substantial impact that online educational content can have in increasing patient awareness and access to medical services. While academic institutions appear effective at highlighting the benefits of WLS, additional effort is needed to provide clearer communication about potential complications to improve patient literacy and understanding of the intervention.

These findings underscore the importance of studying how exposure to online medical content influences patient decision-making, particularly regarding weight-loss treatment options. Given the overall poor quality of WLS videos, evaluating patient understanding of bariatric surgery after consuming online content may clarify how well social media prepares patients for surgical consultation. Conversely, examining why patients accept or decline weight-loss medications may help determine whether the more negative tone of WLM videos contributes to hesitancy or affects attitudes toward pharmacologic treatment.

This study has several limitations. First, the search was conducted at a single point in time; therefore, the first 200 “most relevant” results for each query may have changed since data collection. Additionally, because WLM is a relatively new intervention compared with WLS, emerging long-term data regarding efficacy, complications, and safety continue to evolve and circulate on social media. Governmental health policy changes, insurance coverage expansion for GLP-1 agonists, and increased collaboration between pharmaceutical companies and commercial media entities may influence the tone and framing of subsequent online content. As regulatory guidance and reimbursement structures evolve in the United States, the portrayal of both WLS and WLM on digital platforms may shift accordingly.

Second, although the DISCERN instrument is validated for written materials, some of its criteria may not translate perfectly to video-based content. This mismatch may result in lower scores across both WLS and WLM videos. Third, despite using two independent scorers and an adjudicator, subjective elements remain inherent to both the DISCERN and tone-scoring tools. The DISCERN instrument may also be biased toward informational content over personal narratives, as informational videos more directly address benefits, risks, and alternatives, components heavily weighted in DISCERN, whereas patient experience videos emphasize quality-of-life outcomes that are represented by only a single DISCERN question.

Statistical analyses using Mann-Whitney U, two-way ANOVA, and Kruskal-Wallis tests rely on assumptions such as independence of observations and, for ANOVA, homogeneity of variance. In online video datasets, these assumptions may not be strictly met, potentially affecting p-value precision and interpretation of differences.

In summary, most WLS videos were produced by academic institutions, contained a mix of informational and patient experience content, held a predominantly positive tone, and demonstrated poor overall quality. In contrast, most WLM videos were commercially uploaded, predominantly informational, exhibited less positive and more neutral or negative tone, and demonstrated higher quality. These findings reflect substantial variability in the source, content, tone, and quality of YouTube videos related to weight-loss treatments. They also highlight the need to guide patients toward accurate, balanced educational resources. Future research should explore how viewership of WLS- and WLM-related video content affects patient understanding, attitudes, and treatment choices.

## Data Availability

The datasets analyzed in this study consist of publicly available YouTube videos and can be accessed directly on the platform. Additional details are available from the corresponding author upon reasonable request.
